# Awareness and Disposal Practices of Medicines among the Community in Hawassa City, Ethiopia

**DOI:** 10.1155/2023/4603993

**Published:** 2023-12-06

**Authors:** Daniel Woldamicael Bekele, Elias Dadebo, Girma Tilahun, Zinabu Gebremariam

**Affiliations:** ^1^Hawassa University, Biology Department, Environmental Toxicology Program, Hawassa, Ethiopia; ^2^Hawassa College of Teachers Education, Hawassa, Ethiopia; ^3^Hawassa University, Department of Aquatic Sciences, Fisheries & Aquaculture, Hawassa, Ethiopia

## Abstract

Despite the enormous benefits medicines provide to humanity, their improper disposal frequently leads to detrimental consequences on the environment. Lack of awareness and malpractices concerning expired, leftover, or unused (ELU) medicines have become concerns worldwide. This study assessed community awareness and practices regarding the disposal of ELU medicines in Hawassa City, Ethiopia. A community-based descriptive cross-sectional survey design was used among the urban population of Hawassa City. Multistage sampling procedures were employed to select 405 household (HH) respondents, and purposive sampling techniques were used to select key experts (KEs) and key informants (KIs). A pretested questionnaire was designed for HHs, KEs, and KIs. The results of the study showed that analgesics and antibiotics, used in 52 and 27% of the HHs, respectively, were the most commonly consumed medicines in this city. The vast majority (95.5%) of the HHs did not store expired medicines but disposed of them. Only 10% of the HHs were well informed on how to dispose of ELU medicines. Most (70%) KEs and KIs revealed that there were no awareness-creation mechanisms for the safe disposal of ELU medicines. A significantly high (*p*  <  0.05) percentage (76%) of the HH respondents who were well informed on how to dispose of ELU medicines had higher education, but most (95%) of them indicated that they would not be willing to be involved in “ELU-take-back” programs even if there had been such a mechanism. Field observations confirm significant amounts of medical waste improperly discarded in various areas, including the shores of Lake Hawassa near Hawassa City. The study has shown that awareness of the management of ELU medicines is critically lacking in the community of Hawassa City, posing environmental and human health risks. Moreover, the majority of households practice unsafe disposal of ELU medicines, leading to human health threats and environmental risk.

## 1. Introduction

Medicines have remarkably contributed to enhancing the quality of life [1]. Therefore, they have become indispensable elements in the healthcare system of humans and animals around the globe. Despite their benefits, medicines often bring about adverse effects [2], especially when disposed of into the environment in an unrestrained manner [3].

In the current decade, there is a significant rise in the production and use of medicines globally [4]. However, medicines that go into the hands of people are not all consumed, but large quantities are expired, leftover, or unused (ELU) and finally discarded into the environment [5]. The World Health Organization (WHO) has documented that more than half of the medicines produced worldwide may be prescribed inappropriately [6] as a result they are stored at home and eventually discarded, released into the environment as ELU substances [7]. Therefore, the current situation of environmental drug pollution is attracting increasing public concern about awareness, attitudes, and drug-handling practices from an environmental perspective. To ensure environmental safety and human health, it is imperative that public awareness of ELU drug-handling practices is determined.

In this regard, people in developed countries are generally aware of the consequences of irresponsible disposal of ELU medicines and have put in place well-established systems. However, in most developing countries, the situation is different. There are few research reports that reveal people's awareness and disposal practices of ELU medicines, indicating the existence of an information gap on environmentally friendly disposal practices of ELU medicines. For instance, in Ethiopia, there are only a few reports from the northern and eastern regions of the country. However, in the southern region of the country where this study was conducted, this type of research is nonexistent. To the best of our knowledge, there is no study reported on community perception, attitude, and disposal practice for ELU medicines in Hawassa City, a fast-growing city with nearly half a million inhabitants and intensive human activity. Even worse, the city is located in the vicinity of a lake, which receives all types of liquid and solid waste from the city.

Consequently, Hawassa City deserves such a study for the protection of public and environmental health. Therefore, this study was designed to assess the level of awareness, attitudes, and disposal practices of ELU medicines among households (HHs) in Hawassa City, Ethiopia. The findings of the study will bridge the information gap and shed light on possible mechanisms of handling ELU medicine disposal practices at household levels. Such information can be useful not only to Hawassa City but also to other cities in the country and elsewhere in developing countries.

## 2. Materials and Methods

### 2.1. Study Area

**Figure 1:**
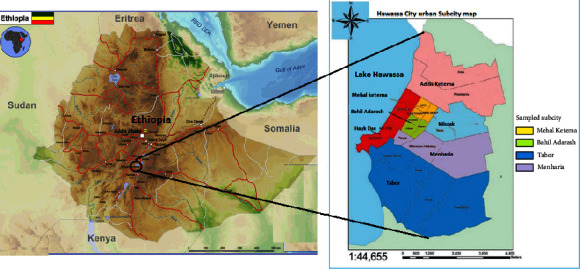
Map of the study area, modified from Eigenmaps [8] and FSM [9].

Hawassa City (Figure 1) is located 275 km South of Addis Ababa, the Ethiopian Capital, in the Rift Valley region, and on the Cairo-Cape-Town Trans-African highway. The city lies within the latitude of 6° 55′ to 7° 6′ N, a longitude of 38° 25′ to 38° 34′ E, and an elevation of 1,708 m.a.s.l. Hawassa City is located in very close proximity to Lake Hawassa, making it a major source of pollution for the lake. Hawassa City is the capital of the Sidama Regional State that consists of seven urban and one rural administrative subcities with a population of more than 450, 000 [10]. The city holds about 153 healthcare facilities, including 5 hospitals, 2 health centers, 31 clinics, 14 diagnostic medical laboratories, 46 stores for medicines, and 55 retail pharmacies [11, 12].

Hawassa City has been among the fastest-growing cities in the country with respect to urbanization and industrial growth with an average urbanization rate of 6.3% per annum for four consecutive years, which is much greater than the national urbanization rate of 4.1% [13].

### 2.2. Study Design and Period

A community-based cross-sectional descriptive survey design, using both qualitative and quantitative methods, was conducted from March 05 to May 30, 2021. A qualitative method was used to make field observations on the waste storage of medicines and disposal practices of households (HHs) using a pre-prepared checklist and photographs. For the quantitative study, prevalidated interviewer-based questionnaires were employed for HHs, Key experts (KEs), and Key informants (KIs). The questionnaires were used to collect information on the types of medicine utilized by the community, occurrences of ELU medicines, storage and disposal practices, and awareness of the community.

### 2.3. Study Population

According to the Hawassa City Administration [12], the city comprises eight subcities (seven urban and one suburban) and 32 “*Kebele*s,” the smallest administrative units. According to the projection of the Ethiopian Central Statistical Agency (CSA), the urban population of Hawassa City in 2021 was 471,952, estimated at a 4% growth rate [13].

### 2.4. Eligibility Criteria

#### 2.4.1. Households

Inclusion criteria were as follows: the seven urban subcities of Hawassa City and their respective 20 “*Kebeles*” (formal settlements with registered house numbers including villas and row houses or condominium housings) were included. Permanent residents of Hawassa City for at least six months were also included. Heads of households and members of households with an age greater than or equal to 18 years old were included and at least one member, who is mentally healthy and willing to participate, was included in the study.

Exclusion criteria: one subcity (Tula) and each of its 12 “*Kebeles*” were excluded from the study population because they are suburban areas. Informal settlements, huts, and dormitories were excluded. Residents who attended Hawassa City for the first time within six months, closed houses during data collection for three consecutive days, and residents who were unable to be communicated or interviewed were excluded from the study.

#### 2.4.2. Key Experts and Key Informants

Inclusion criteria were as follows: individuals with expertise in the fields of pharmacy, public health, and environmental protection were included as key experts (KEs). In addition, waste management workers were included as key informants (KIs). The KEs included in this study had a minimum of a bachelor's degree and five years of professional experience in their respective fields, with at least one year of practical experience specifically within Hawassa City. Pharmacists employed at highly visited pharmacies, with a visitor count exceeding 100 per day, and public health and environmental protection professionals employed in Hawassa City Environmental Protection Agency or health centers in Hawassa City were also included in the study. The KIs (waste management manual workers) included in this study had a minimum of three years of work experience in waste collection or as an employee associated with waste management activity at a waste disposal site within Hawassa City.

#### 2.4.3. Field Observations

Inclusion criteria were as follows: the field observations included sampled HH garbage bins that were only informed by HH respondents of the presence of fresh medicine waste during data collection. Ditches, drainage lines, roadsides, and the shorelines of Lake Hawassa, adjacent to the sampled “*Kebele*,” and the waste disposal site designated by the Hawassa City Municipality were included in the field observation.

Exclusion criteria: the field observations did not include waste transfer stations, hospitals, clinics, health center waste collection bins, and storage sites at any premises.

### 2.5. Sample Size Determination and Sampling Techniques

#### 2.5.1. Households

The sample size was determined using the single population proportion formula in equation (1) as described by Kothari [14]. As there was no previous study conducted in Hawassa City, a sample proportion of 50% was considered suitable in the absence of any existing data in the study area [14]. Therefore, to calculate the required sample size, a 95% confidence level, 5% margin error, and 50% chance of respondents agreeing to the study were applied.

(1) $$\begin{array}{c} N=\frac{Z^{2}*p*q}{d^{2}}, \end{array}$$

where *N* is the minimum sample size, *z* is the level of confidence according to the normal standard distribution which corresponds to the 95% confidence interval (*z* = 1.96), p is a proportion of 50% (0.5), *q* = 1 − *P*, and *d* is the desired degree of accuracy or tolerated margin of error which is 5% (0.05).

The study employed a multistage sampling approach, incorporating various methods to ensure a representative sample. The subcities and *Kebeles* were selected using a simple random sampling method. A *Kebele* is the smallest administrative unit in Ethiopia. The sample size of HHs in each *Kebele* was determined using a proportional allocation method. In addition, a systematic random sampling technique was employed to identify the housing units.

A total of eight *Kebeles* were randomly chosen for inclusion in the study, with two *Kebeles* selected from each of the four subcities (as shown in Table 1). Given the homogeneity of the population, a 5% compensation for nonresponse was incorporated, resulting in a final sample size of 405 households. The proportional allocation technique (as per equation (2)) was employed to allocate the 405 households among the eight *Kebeles* that were selected, as depicted in Table 1.

(2) $$\begin{array}{c} \text{ni}=\left[\frac{\text{Ni}}{N}\right]*n, \end{array}$$

where ni is the sample size for each *Kebele*, Ni is the total HHs in each *Kebele*, *N* is the total HHs of each *Kebele,* and *n* is the sample size.

The study units (each HH included in the study) were selected based on a systematic random sampling technique using the *n*^th^ interval. Ni and ni are the total number of households in the *Kebele* and the number of households included in the study from that *Kebele,* respectively.

#### 2.5.2. Key Experts, Key Informants, and Field Observations

Key experts (KEs) and key informants (KIs) were deliberately chosen, and specific field observation sites were designated with the intention of acquiring a comprehensive understanding of the study. This approach aimed to triangulate the results obtained from the household (HH) survey. It included 15 pharmacists, 5 health officers, 3 environmental protection experts, 2 environmental and public health experts as KEs, and 5 waste management manual workers as KIs (Table 2).

Four sites were also purposively selected for field or onsite observations. These included HH garbage bins, the city solid waste disposal sites, ditches, roadsides, and lake shores located adjacent to the sampled *Kebele*s.

### 2.6. Data Collection Tool

Data collection was carried out by using an interviewer-based questionnaire for HHs, KEs, and KIs, and an observational checklist was prepared for field observations.

#### 2.6.1. Households

The HH questionnaire included a consent request, socioeconomic information, and 18 close-ended survey questions. The consent notes in the questionnaire requested the agreement of 18 years old and above members of the household to participate in the study. Part one of the questionnaire was about the respondents' personal information including gender, age, marital status, family size, educational status, and family income. Part two included questions on classes of medicines most utilized by HHs. Part three of the questionnaire was on the occurrence of ELU medicines. Part four included questions on the awareness and attitude of respondents on the disposal of ELU medicines on a 5-point Likert scale. Part five included questions on the disposal practices of the HHs. In this last section, the respondents were required to indicate the phrases that best described their usual practices.

#### 2.6.2. Key Experts and Key Informant Interview

Questions for key experts (KEs) and key informants (KIs) were developed based on the main findings of the HH questionnaire in this study. The questionnaire consisted of a total of 10 questions, 8 closed-ended questions and 2 open-ended questions for KE, and 5 closed-ended questions for KI.

#### 2.6.3. Field Observations

A checklist was developed to document the occurrence and classification of ELU medicines at designated locations. Using this checklist and field cameras, we thoroughly examine the presence or absence of medications in household garbage bins, solid waste disposal sites, ditches/roadsides, and lakeshores.

### 2.7. Validation of the Instruments

#### 2.7.1. Content Validation

The HH questionnaire was adopted from other studies [15–17], adapted to the local context in English, and translated into Amharic to obtain valid responses from the respondents. A bilingual expert (Amharic and English) performed the forward translation, and another bilingual expert independently performed the backward translation. The original and translated questionnaires were thoroughly reviewed and discussed by the research team, and items that needed corrections were accordingly made to the Amharic version. To assess the relevance of each item in the questionnaire, a panel of ten pharmacists, including six from academia and four from other sectors, evaluated and scored each item on a four-point Likert scale, ranging from 1 (irrelevant) to 4 (excellent). The content validity index, as proposed by Lynn [18], was used to calculate the validity of the questionnaire. Based on the suggestions provided by the experts, changes were made to the questionnaire. Two questions were removed and eight were modified, and in the survey, based on the suggestions from the experts, the number of questions of the Amharic version was finally brought up to 18. The Amharic version of the questionnaire was pretested by administering it to 30 households whose age was 18 and above in a nonselected “Kebele” of the Hawassa City. Any ambiguities or other questions raised by the respondents were noted, and modifications of questions, as well as their anticipated responses, were revised to ensure the generation of accurate responses.

Similarly, the KE and KI questionnaires were reviewed by experts in the field and pretested on 10 randomly selected pharmacists. All the pretest results were not included in the actual study.

#### 2.7.2. Reliability or Internal Consistency

The internal validity of the research tool was maintained by reviewing different parts of the research domain. Based on this, Cronbach's alpha test was conducted to determine the internal consistency and reliability of the HH questionnaire items. For each item, a Cronbach alpha coefficient greater than 0.5 was considered acceptable [17]. As shown in Table 3, the first item (socioeconomic characteristics) was 0.72, the second element (occurrence) was 0.57, the third item (awareness/attitude) was 0.67, and the fourth item (practice) was 0.61. Therefore, the values obtained from the test were acceptable to achieve the objectives of the study.

### 2.8. Data Collection Method

#### 2.8.1. Households

Upon validation of the tools, the trained data collectors, who were students from Hawassa University, proceeded to collect data from all selected households. The interviewers provided a comprehensive explanation of the survey's purpose, ensured the participants of their data's anonymity and confidentiality, and exclusively recruited participants who met the inclusion criteria and provided consent to participate in the study. The interviewers conducted the interview by utilizing the Amharic version of a structured questionnaire.

#### 2.8.2. Key Experts/Key Informants and Field Observations

The researchers collected data from KEs and KIs. Following a thorough explanation of the study's purpose, participants who met the inclusion criteria were exclusively recruited to provide their opinions. In addition, the researcher personally conducted field observations. The checklist used for this purpose examined the presence of pharmaceutical waste, including tablets, capsules, syrups, ointments, fluids, and empty containers such as medicine bottles, packages, and intravenous (IV) tubes/catheters.

### 2.9. Data Processing and Analysis

All statistical analyses were performed using the Statistical Package for Social Science (SPSS) version 24 and a Microsoft Excel spreadsheet. The completeness and consistency of the data were verified and then entered into an Excel spreadsheet. Subsequently, the cleaned data were organized, coded, summarized, and transferred to SPSS. Categorical variables were presented as frequency (percentage). To evaluate the difference in awareness, attitude, and disposal practices of expired, leftover, and unused (ELU) medicines, a chi-square test (*χ*^2^) was employed. Statistical significance was determined for different socioeconomic groups of the household (HH) respondents in Hawassa City at *p*  <  0.05.

## 3. Results

### 3.1. Socioeconomic Status of Respondents

#### 3.1.1. Households

The socioeconomic status of the household (HH) respondents is presented in Table 4. Out of the total of 405 HHs that were sampled, 402 responded to the questionnaire, resulting in a response rate of 99.3%. Among these respondents, 66.2% were females, while the remaining 136 (33.8%) were males. The age group with the highest number of respondents was 25–31 years, accounting for 138 (34.3%) individuals, followed by the age group of 18–24 years, which consisted of 79 (19.7%) individuals. Approximately 79% of the respondents were married. The two largest family size groups among the respondents were those with family sizes of 2–4 (45.0%) and 5–10 (42.0%). Nearly half of the respondents, 196 (48%), were college graduates. In terms of monthly family income, approximately one-third of the respondents, 142 (35.3%), earned between 5 and 10 thousand Ethiopian Birr (ETB) (equivalent to 125 to 250 US dollars), while 135 respondents (33.6%) earned less than 5 thousand ETB (125 US dollars) per month.

### 3.2. Classes of Medicines Most Utilized by Households

#### 3.2.1. Households

**Figure 2:**
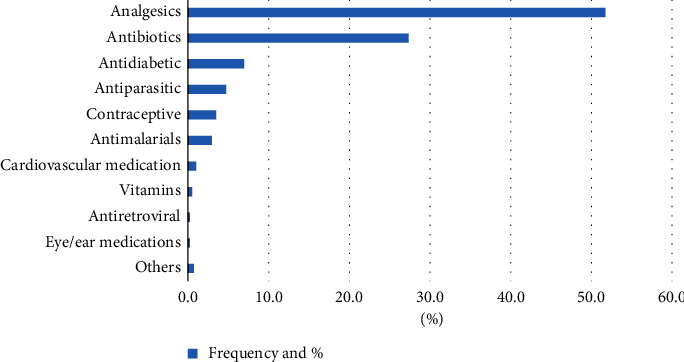
Percentage frequency of medicines most utilized by HH respondents in Hawassa City (*N* = 402).

Figure 2 presents the most frequently employed types of medications by households (HHs) in Hawassa City. Of the total HH respondents, 52% utilized analgesics, while 27% used antibiotics. This indicates that these two categories of medications were the most commonly utilized by the community in Hawassa City. In addition, other classes of medications utilized by the community of Hawassa City included antidiabetic medications, which were consumed by 7% of the HHs, antiparasitic drugs (4.7%), contraceptives (3.5%), antimalarial drugs (3%), and cardiovascular medication (1%). The remaining 1.6% of medications taken by the community comprised vitamins, antiretroviral medicines, and eye and ear drops, among others.

#### 3.2.2. Key Experts

**Figure 3:**
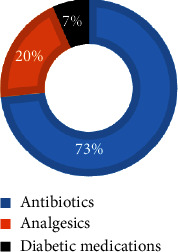
Types of medicines mostly purchased by the community of Hawassa City (pharmacist-KEs, *N* = 15).

Based on the responses of eleven out of fifteen pharmacist-KEs, nearly three-quarters (73%) of the Hawassa City community purchased antibiotics. In contrast, 20% of the community purchased analgesics, while 7% purchased diabetic medications (Figure 3).

### 3.3. Occurrence of Expired, Leftover, or Unused Medicines

#### 3.3.1. Households

Table 5 presents the community's responses regarding the occurrences and quantities of expired, leftover, or unused (ELU) medicines. The data reveal that the vast majority of household (HH) respondents, specifically 384 individuals (95.5%), reported that they promptly dispose of expired medicines instead of storing them. Conversely, only 18 HH respondents (4.5%) admitted to storing expired medicines at home. Out of these 18 respondents, the majority (11 out of 18) stored 1 to 3 doses of expired medicines, while the remaining stored 4 to 6 doses.

Furthermore, according to item 3 in Table 5, it was found that 65 HHs (16.2%) stored leftover or unused medicines at home. Among these households, 75% stored 1 to 3 doses of medicines, while the remaining 25% stored 4 to 6 doses. The stored ELU medicines were categorized into nine classes, as indicated in item 5 of Table 5. Analgesics accounted for slightly over half of the stored ELU medicines, followed by antibiotics (25%), cardiovascular medications (6%), and antidiabetics (6%). The remaining categories included antiphrastic medicines (3.6%), contraceptives (2.4%), antimalarial medicines (2.4%), topical medicines (1%), and eye drops (1%).

#### 3.3.2. Field Observations

During field observations, various medical supplies such as tablets, capsules, syrups, ointments, medical gloves, syringes, needles, medicine bottles, and intravenous (IV) tubes/catheters were commonly observed in the area. Notably, freshly discarded analgesics and antibiotics were found in sampled HH garbage bins. Analgesics such as Advil (ibuprofen), Aleve (naproxen), and nonsteroidal anti-inflammatory drugs (NSAIDs), along with Gofen, were more prevalent during the field visits compared to antibiotics.

### 3.4. Community Awareness and Attitude

#### 3.4.1. Households

The findings of this study indicate that only about 10% of HHs reported being well-informed or adequately informed about the proper disposal of expired or unused (ELU) medicines (Table 6). Conversely, 50% of respondents were either poorly informed or lacked any information on the subject, while approximately 40% held a neutral. Item 2 of Table 6 reveals that approximately 47% of household respondents disagreed or strongly disagreed with the appropriateness of their current disposal practices. In contrast, nearly 43% of HHs expressed agreement with the suitability of their current disposal methods. Only around 11% of HH respondents admitted to being uncertain about the appropriateness of their current disposal practices. Regarding the community's willingness to participate in a “medicine-take-back” program for ELU medicines, a significant majority (80.6%) of HHs expressed a willingness to hand over such medicines to designated locations (Item 3 of Table 6). However, approximately 10% of households stated that they would not be willing to return ELU medicines, and 11% remained neutral on the matter.

#### 3.4.2. Key Experts and Key Informants

**Figure 4:**
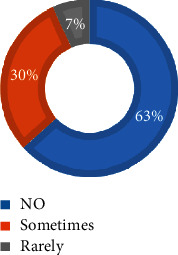
Assessment of awareness creation on safe disposal mechanisms of medicines among HHs in Hawassa City (KEs and KIs, *N* = 30).

As depicted in Figure 4, a significant majority of the KEs and KIs, amounting to 70%, indicated the absence of any mechanisms for raising awareness regarding the safe disposal of medicines among HHs. On the other hand, 30% of the KEs and KIs acknowledged the occasional provision of awareness-creation initiatives.

### 3.5. Association between the Socioeconomic Profile and the Awareness and Attitude of the Community

Table 7 presents the results of the chi-square test conducted to examine the relationship between the socioeconomic profile of the respondents and the awareness and attitude of the community towards the disposal of ELU medicines. The findings indicate that the educational level of the HH respondents was significantly associated (*p*  <  0.05) with the community's awareness of how to dispose of ELU medicines (*χ*^2^ (2, *N* = 364) = 10.22, *p* = 0.006). In addition, the educational level and family size of the respondents were significantly associated with the appropriateness of their current ELU disposal mechanism (*χ*^2^ (2, *N* = 364) = 26.50, *p* = 0.001 and *χ*^2^ (1, *N* = 402) = 13.69, *p* = 0.0002), respectively. Furthermore, the educational level and family income of the respondents were significantly associated (*p*  <  0.05) with the community's willingness to participate in the “ELU-take-back” program, whenever available (*χ*^2^ (2, *N* = 364) = 8.28, *p* = 0.01 and *χ*^2^ (2, *N* = 402) = 6.77, *p* = 0.03).

The post hoc analysis revealed that a significantly higher percentage (76%) of HH respondents with college and above educational levels were either well informed or just informed on how to dispose of ELU medicines (*χ*^2^ (1, *N* = 364) = 9.06, *p* = 0.003). Moreover, 86% of HH respondents with college and higher educational levels disagreed or strictly disagreed with the appropriateness of their current ELU disposal habits (*χ*^2^ (1, *N* = 364) = 26.01, *p* = 0.001). However, 95% of college and above educational level and 74% of high family income HH respondents were not willing or strictly not willing to participate in the “ELU-take-back” program if there had been one (*χ*^2^ (1, *N* = 364) = 7.84, *p* = 0.005 and *χ*^2^ (1, *N* = 402) = 49, *p* = 0.0001), respectively.

### 3.6. Community Disposal Practices of Expired, Leftover, or Unused Medicines

#### 3.6.1. Household

Table 8 shows community responses to current ELU drug disposal practices. More than two-thirds (68%) of the 272 HH respondents disposed of expired medications in the household garbage bins. Approximately 1 in 5 respondents (21%) said they flushed expired medication down the toilet. None of the respondents reported that they would not want to utilize the “medicine-take-back” option, if the option had existed. Only a small portion of the population (4%) buried expired medicines underground. The majority (75%) of HH respondents disposed of leftover and unused medicines in the same manner, while the rest (25%) stored the medicines until their expiry dates.

#### 3.6.2. Key Experts

The awareness of the KEs on the disposal practices of the community on ELU medicines is given in Table 9. All KEs unanimously confirmed the absence of designated collection points for ELU medicines within the city. Furthermore, a significant majority (84%) of the KEs expressed a lack of knowledge, on their part, regarding the issue of the “medicine-take-back” program. In addition, more than half (56%) of the KEs stated that they were certain of the fact that there was no medicine-supplying agent that collected back ELU medicines from the community of Hawassa City. The rest 44% of the KEs did not know of the existence of any medicine-supplying agents that would collect ELU medicines from the community.

#### 3.6.3. Field Observations

The field observations showed a significant abundance of medical waste being haphazardly and thoughtlessly discarded at municipal solid waste disposal sites, ditches, roadsides, and the shores of Lake Hawassa. Among the identified discarded medical items were syrup bottles, plastic bags containing tablets, used condoms, intravenous (IV) tubes, and strips of capsules with residual capsules.

### 3.7. Association between Socioeconomic Profile and Medicine Disposal Practices of the Community

The association between the socioeconomic profile of HH respondents and ELU medicine disposal practices was analyzed, and the results are presented in Table 10. The results of the chi-square test indicate that there is no statistically significant association (*p*  >  0.05) between the socioeconomic status of the respondents, including gender, age, family size, educational status, and family income, and their disposal practices of expired medicines. However, it was found that the family size and educational status of the HH respondents were significantly associated (*p*  <  0.05) with the disposal practices of leftover or unused medicines. The chi-square test results for family size (*χ*^2^ (1, *N* = 402) = 8.13, *p* = 0.004) and educational status (*χ*^2^ (1, *N* = 364) = 8.32, *p* = 0.004) indicate a significant relationship with the disposal practices of ELU medicines.

## 4. Discussion

Reliable information on the awareness and practice of households with respect to the disposal of ELU medicines in a community should depend on the willingness of the residents to participate in the information-gathering process for studies. The high response rate of the community (99.3%) in this study is indicative of the community's willingness to participate in the study without any incentives or obligatory measures. We believe that data collected from such respondents, as in this study, should be reasonably reliable and that the conclusions drawn from there are valid. Our study findings reveal that the most commonly consumed and widely identified ELU medicines were analgesics, antibiotics, and diabetic medications. This may be attributed to the fact that medicines for mild illnesses can be easily obtained over the counter without prescriptions. In addition, regularly taken drugs such as diabetic medications can be purchased using refill prescriptions that are repeatedly presented. This finding is consistent with a study conducted in Jordan [17], where self-medication was practiced for mild illnesses due to the high costs of medical consultations and treatments. A similar global trend has been reported in Makati Medical Center (MMC), Makati, Metro Manila, Philippines [19], with hydrocodone (an analgesic), metformin (an antidiabetic), and various antibiotics being the most commonly prescribed drugs worldwide. This suggests that these three types of drugs are probably the most commonly consumed around the world.

The majority of the community did not store ELU medicines in their homes but instead disposed of them immediately. Although this practice is not ideal, it serves to protect vulnerable family members from accidental poisoning. The study revealed improper disposal practices of ELU medicines, including disposal in household garbage bins or flushing them down the toilets, with no consideration for the bioactive characteristics of the medicines. These practices were widely observed during field observations, where various medicines were found disposed of on street corners, drainage lines, disposal sites, and the shores of Lake Hawassa. In addition, discarded medical items, such as used intravenous (IV) tubes, were also detected during field observations, indicating that healthcare centers, such as hospitals and clinics, do not dispose of medical supplies properly. The disposal of these medicines into the environment may cause the active ingredients to leach into the soil and water bodies, potentially contaminating the surrounding ecosystem. Such practices may also produce cocktails of medicines that pose threats to human health.

A significant proportion of the community has demonstrated a willingness to actively participate in the effective management of ELU medicines. The response of the community indicates that there is a favorable environment to commence the ELU “medicine-take-back” program within the community of Hawassa City. Such a positive response may rectify the current situation, provided that other appropriate measures such as the implementation of “ELU-take-back” programs are initiated. The willingness of the community to engage in the management of ELU medicines, as observed in this study, is consistent with findings from a study conducted in Saudi Arabia, where 90% of respondents expressed their willingness to partake in a “medicine-take-back” program [20]. Similarly, Bekker et al. [21] from the Netherlands reported a similar outcome.

On the contrary, a significant proportion of educated individuals, who had attained college-level education or higher, exhibited reluctance to participate in the “ELU-take-back” programs, even if they were available. This is unexpected because those with the highest levels of education should be most aware of environmental issues. Moreover, this reluctance among the educated community members is inconsistent with various reports [16, 17]. As we did not hold focus group discussions, we cannot conclude the reasons for this, but there is a possibility that some form of incentives to encourage their participation in such programs should be taken into account.

This study has revealed that there is a lack of adequate information, in the study area, on safe disposal practices and malpractices in discarding ELU medicines, which has also been reported in various parts of the world, including Nigeria [22], Ghana [23], India [24], and Saudi Arabia [25]. The predominant disposal system for ELU medicines has been reported to be garbage bins in Qatar [26], the Western Kingdom of Saudi Arabia [27]. However, the percentage of households that dispose of ELU medicines in garbage bins in this study was lower than those reported in Nigeria [28], Afghanistan [15], and Cyprus [29]. This suggests that the direct contamination of the environment by ELU medicines in this study is relatively low, and therefore, its direct impact on the ecology is minimal. However, ELU medicines disposed of in garbage bins ultimately end up in landfills or municipal solid waste disposal sites, with the potential to contaminate the environment, particularly surface and groundwater sources [30].

The present study is subject to certain limitations as we did not conduct focus group discussions (FGDs). These constraints pertain specifically to the absence of “medicine-take-back” or other alternative programs for the collection of expired, leftover, or unused (ELU) medicines in Hawassa City. Had we conducted FGD, we could have gained insight into the role of health professionals, through their communication and consulting activities, to support the community in improving adherence to the use or proper disposal of ELU medicines. It would have also been helpful in drawing conclusions on the reasons why safe ELU disposal has not been introduced to the community of Hawassa City, and its future prospects as a policy issue at the national level. In addition, as this is the first report from the current study area, we did not have comparative data to address possible seasonal variations. This calls for further studies that will overcome the limitations of this study and, if possible, be accompanied by an investigation into the detailed reasons leading to the emergence of ELU medicines at the household level.

## 5. Conclusions

This study has revealed that although the community in Hawassa City, Ethiopia, demonstrated a potentially responsive attitude, it exhibited a considerably low level of awareness and environmentally unsafe disposal practices concerning expired, leftover, or unused (ELU) medicines.

Therefore, it is imperative to implement measures for raising awareness on safe disposal techniques to enhance community consciousness. However, such measures should not be confined solely to households but should also include healthcare facilities. Retail pharmacies and drugstores must strictly control the sale of medicines without prescriptions to limit the possession of medicines surplus to requirements that would lead to ELU medicines and ultimately result in unsafe disposal. The findings from our study will contribute to reducing the existing information gap and shed light on possible mechanisms to manage ELU drug-handling practices at the household level in Hawassa City.

In a nutshell, despite the limitations, our study remains one of the very few studies conducted in Ethiopia that provides valuable insights into the attitude, perception, and disposal practices of ELU medicines. Moreover, the possible contribution of this study to understanding disposal practices of ELU medicines in other cities in Ethiopia and elsewhere in the developing countries cannot be discounted.

## Figures and Tables

**Table 1:** Total number of households in each sampled *Kebele* and the respective sample size included in the study.

Subcities	*Kebeles*	Total number of HHs in each *Kebele* (Ni)	The number of HHs included in the study (ni)
Mehal-Ketema	Addis Ababa	1698	62
	Nigat-Kokeb	1908	70

Bahil-Adarsh	Andinet	753	28
	Adare	822	30

Tabor	Alamura	970	35
	Dume	2170	80

Menaheria	Gue-stadium	1680	62
	Millennium	1036	38

Total		*N* = 11037	*n* = 405

**Table 2:** Professions and institutions of the key experts (KEs) and key informants (KIs) involved in the study.

Professions	Institutions	Role	Number
Pharmacist	Private pharmacy	KEs	12
	Government hospital	KEs	2
	Private hospital	KEs	1

Health officer	Health center	KEs	5

Environment protection expert	Environmental Protection Agency	KEs	3

Environment and public health expert	City municipality	KEs	2

Waste management manual worker	City municipality	KIs	5

**Table 3:** Reliability statistics.

Items	Cronbach's alpha	Cronbach's alpha based on standardized items	N of items
Socioeconomic characteristics	0.719	0.583	6
Most utilized class and occurrence	0.571	0.501	6
Awareness and attitude	0.673	0.938	3
Disposal practices	0.613	0.609	3

**Table 4:** Socioeconomic profile of household respondents.

Variables		HHs (*N* = 402) frequency (%)
Gender	Male	136 (33.8)
	Female	266 (66.2)

Age (years)	18–24	79 (19.7)
	25–31	138 (34.3)
	32–38	70 (17.4)
	39–45	32 (8.0)
	46–52	27 (6.7)
	53–59	38 (9.5)
	>59	18 (4.5)

Marital status	Married	317 (78.9)
	Unmarried	81 (20.1)
	Divorced	4 (1.0)

Family size (number)	1 (single)	49 (12.2)
	2–4	181 (45.0)
	5–10	169 (42.0)
	11–15	3 (0.7)

Educational level	Only read and write	38 (9.5)
	Grade 1–8	45 (11.2)
	Grade 9–12	42 (10.4)
	Above high school, first-degree,	196 (48.8)
	Second-degree, and above	81 (20.1)

Family monthly income, ETB	<1,000	3 (0.7)
	1000–5000	135 (33.6)
	5001–10000	142 (35.3)
	10001–20000	74 (18.4)
	20001–40000	41 (10.2)
	>40000	7 (1.7)

ETB- Ethiopian Birr.

**Table 5:** Occurrences and quantities of expired, leftover, or unused medicines at HHs.

No	Items		(*N* = 402) HHs frequency (%)
1	Occurrence of expired medicines	Yes	18 (4.5)
		No	384 (95.5)

2	Quantity in doses of leftover or unused medicines	1–3	11 (61.1)
		4–6	7 (38.9)

3	Occurrence of leftover or unused medicines	Yes	65 (16.2)
		No	337 (83.8)

4	Quantity in doses of leftover or unused medicines	1–3	50 (76.9)
		4–6	15 (23.1)

5	Type(s) of expired, leftover. or unused medicine(s)	Analgesics	43 (51.81)
		Antibiotics	21 (25.3)
		Cardiovascular medication	5 (6.02)
		Antidiabetics	5 (6.02)
		Antiphrastic	3 (3.61)
		Contraceptive	2 (2.41)
		Antimalarial	2 (2.41)
		Topical/cream	1 (1.2)
		Eye drop	1 (1.2)

**Table 6:** Awareness and attitude of the community on disposal of medicines.

No	Items		HHs *N* = (402) frequency (%)
1	Getting information on how to dispose of medicine waste	Well informed	8 (2.0)
		Informed	31 (7.7)
		Neutral	161 (40.0)
		Less informed	83 (20.7)
		No information	119 (29.6)

2	Agreement on the appropriateness of the current disposal mechanism	Strongly agree	47 (11.7)
		Agree	124 (30.8)
		Neutral	43 (10.7)
		Disagree	118 (29.4)
		Strongly disagree	70 (17.4)

3	Willingness to deliver ELU medicines when a specific location is arranged	Strongly willing	147 (36.6)
		Willing	177 (44.0)
		Neutral	36 (9.0)
		Unwilling	39 (9.7)
		Strictly unwilling	3 (0.7)

**Table 7:** The association between the socioeconomic profile of the respondents and their awareness and attitude on the disposal of ELU medicines.

Variable	Frequency (%)	Gender		Age (year)		Family size (number)		Educational^a^ level		Family income (ETB)	
		Male	Female	≤35	>35	≤4	>4	Grade 1–12	College & above	≤5000	>5000
*Do you have information on how to dispose of ELU medicines (N* *=* *402)*											
Well informed/informed	38 (9.5)	14 (36.8)	24 (63.2)	25 (65.8)	13 (34.2)	22 (57.9)	16 (42.1)	9 (23.7)	29 (76.3)	16 (42.1)	22 (57.9)
Neither informed/nor less informed	161 (40.0)	59 (36.6)	102 (63.4)	100 (62.1)	61 (37.9)	90 (55.9)	71 (44.1)	22 (15.4)	121 (84.6)	49 (30.4)	112 (69.6)
Less informed/no information	203 (50.5)	63 (31)	140 (69)	147 (72.4)	56 (2.6)	118 (58.1)	85 (41.9)	56 (30.6)	127 (69.4)	73 (36.3)	130 (63.7)
		*χ* ^2^ = 1.43, *p* = 0.48		*χ* ^2^ = 4.42, *p* = 0.11		*χ* ^2^ = 0.19, *p* = 0.9		*χ* ^2^ = 10.22, *p* = 0.006		*χ* ^2^ = 1.88, *p* = 0.39	

*Do you agree that the disposal mechanism you follow is appropriate? (N* *=* *402)*											
Strongly agree/agree	173 (43)	56 (32.4)	117 (67.6)	117 (67.6)	56 (32.4)	89 (51.4)	84 (48.6)	59 (36.9)	101 (63.1)	69 (40.4)	104 (59.6)
Neither agree/nor disagree	43 (10.7)	13 (30.2)	30 (69.8)	32 (74.4)	11 (25.6)	36 (83.7)	7 (16.3)	4 (11.8)	30 (88.2)	16 (37.2)	27 (62.8)
Strictly disagree/disagree	186 (46.3)	67 (36)	119 (64)	123 (66.1)	63 (33.9)	105 (56.5)	81 (43.5)	24 (14.1)	146 (85.9)	53 (29.3)	133 (70.7)
		*χ* ^2^ = 0.81, *p* = 0.66		*χ* ^2^ = 1.09, *p* = 0.57		*χ* ^2^ = 14.73, *p* = 0.01		*χ* ^2^ = 26.50, *p* = 0.001		*χ* ^2^ = 4.84, *p* = 0.08	

*If the government has arranged a specific location for collecting expired/unused medicines, are you willing to hand over? (N* *=* *402)*											
Strongly willing/willing	322 (80.1)	108 (33.5)	214 (66.5)	220 (68.3)	102 (31.7)	188 (58.4)	134 (41.6)	75 (25.4)	220 (74.6)	108 (34.2)	214 (65.8)
Neither willing/nor not willing	36 (9)	13 (36.1)	23 (63.9)	21 (58.3)	15 (41.7)	16 (44.4)	20 (55.6)	10 (31.2)	22 (68.8)	19 (52.8)	17 (47.2)
Strictly not willing/not willing	44 (10.9)	15 (34.1)	29 (65.9)	31 (70.5)	13 (29.5)	26 (59.1)	18 (40.9)	2 (5.4)	35 (94.6)	11 (25.6)	33 (74.4)
		*χ* ^2^ = 0.09, *p* = 0.95		*χ* ^2^ = 1.65, *p* = 0.43		*χ* ^2^ = 2.64, *p* = 0.26		*χ* ^2^ = 8.28, *p* = 0.01		*χ* ^2^ = 6.77, *p* = 0.03	

^a^(*N* = 364); thirty-eight respondents were excluded from the educational level in this study, on the ground that their level of education was out of the given range.

**Table 8:** Household disposal practices of expired, leftover, or unused medicines.

Items		HHs (*N* = 402) frequency (%)
Disposal of expired medicines from HHs	Put them in the garbage	272 (67.7)
	Flush them down the toilet	83 (20.6)
	Burn them	31 (7.7)
	Bury underground	16 (4)
	“Medicine-take-back”	0 (0)

Unexpired but leftover/unused medicines in HHs	Dispose of them just like the expired medicines	301 (74.9)
	Keep them until they expired	101 (25.1)

**Table 9:** Key experts' awareness on the disposal of ELU medicines in the HHs of Hawassa City.

Items		KEs (*N* = 25) frequency (%)
Presence of designated medicine-waste collection point/s?	Yes	0
	No	25 (100)

Prior knowledge of the “medicine-take-back” system/program	Yes	4 (16)
	No	21 (84)

Medicine wholesalers/distributors/pharmacies collect back ELU medicines from customers	No	14 (56)
	Do not have information	11 (44)

**Table 10:** Associations between respondents' socioeconomic status and practices of ELU medicine disposal.

Variable	Frequency (%)	Gender		Age (year)		Family size (number)		Educational^a^ status		Family income (ETB)	
		Male	Female	≤35	>35	≤4	>4	Grade 1–12	College & above	≤5000	>5000
*How do you dispose of expired medicines? (N* *=* *402)*											
Throw them in the garbage	272 (66.5)	97 (35.7)	175 (64.3)	184 (67.6)	88 (32.4)	155 (57.0)	117 (43.0)	57 (22.9)	192 (77.1)	90 (33.1)	182 (66.9)
Bury underground	16 (4.1)	7 (43.8)	9 (56.3)	11 (68.8)	5 (31.3)	12 (75)	4 (25)	2 (16.7)	10 (83.3)	1 (6.3)	15 (93.8)
Throw/flush them in the toilet	83 (21.4)	23 (27.7)	60 (72.3)	53 (63.9)	30 (36.1)	46 (55.4)	37 (44.6)	19 (25.7)	55 (74.3)	34 (41)	49 (59)
Burn them	31 (8.0)	9 (29.0)	22 (71.0)	24 (77.4)	7 (22.6)	17 (54.8)	14 (45.2)	9 (31)	20 (69)	13 (41.9)	18 (58.1)
		*χ* ^2^ = 2.79, *p* = 0.42		*χ* ^2^ = 1.91, *p* = 0.59		*χ* ^2^ = 2.25, *p* = 0.52		*χ* ^2^ = 1.24, *p* = 0.74		*χ* ^2^ = 7.70, *p* = 0.05	

*What do you do with unexpired but leftover/unused medicines? (N* *=* *402)*											
Keep them until they expire	101 (25.1)	37 (36.6)	64 (63.4)	75 (74.3)	26 (25.7)	70 (69.3)	31 (30.7)	10 (11.8)	75 (88.2)	32 (31.7)	69 (66.3)
Dispose them as expired medicine	301 (74.9)	99 (32.9)	202 (67.1)	197 (65.4)	104 (34.6)	160 (53.2)	141 (46.8)	77 (27.6)	202 (72.4)	106 (33.1)	195 (66.9)
		*χ* ^2^ = 0.41, *p* = 0.51		*χ* ^2^ = 3.01, *p* = 0.82		*χ* ^2^ = 8.13, *p* = 0.004		*χ* ^2^ = 8.32, *p* = 0.004		*χ* ^2^ = 0.40, *p* = 0.524	

^a^(*N* = 364); thirty-eight respondents were excluded from the educational status in this study because their levels of education were out of the given range.

## Data Availability

The data used to support the findings of the study are available from the corresponding author upon request.
